# A Model of Chronic Exposure to Unpredictable Mild Socio-Environmental Stressors Replicates Some Spaceflight-Induced Immunological Changes

**DOI:** 10.3389/fphys.2018.00514

**Published:** 2018-05-09

**Authors:** Fanny Gaignier, Christine Legrand-Frossi, Emilien Stragier, Julianne Mathiot, Jean-Louis Merlin, Charles Cohen-Salmon, Laurence Lanfumey, Jean-Pol Frippiat

**Affiliations:** ^1^Stress Immunity Pathogens Laboratory, EA7300, Faculty of Medicine, Université de Lorraine, Vandœuvre-lès-Nancy, France; ^2^INSERM UMR894, Centre de Psychiatrie et Neuroscience, Paris, France; ^3^Institut de Cancérologie de Lorraine, Service de Biopathologie and CNRS UMR 7039 CRAN, Université de Lorraine, Vandœuvre-lès-Nancy, France; ^4^INSERM U1141, PROTECT, Université Paris Diderot, Sorbonne Paris Cité, Hôpital Robert Debré, Paris, France

**Keywords:** mouse model, spaceflight, stress, lymphocytes, antibodies, cytokines

## Abstract

During spaceflight, astronauts face radiations, mechanical, and socio-environmental stressors. To determine the impact of chronic socio-environmental stressors on immunity, we exposed adult male mice to chronic unpredictable mild psychosocial and environmental stressors (CUMS model) for 3 weeks. This duration was chosen to simulate a long flight at the human scale. Our data show that this combination of stressors induces an increase of serum IgA, a reduction of normalized splenic mass and tends to reduce the production of pro-inflammatory cytokines, as previously reported during or after space missions. However, CUMS did not modify major splenic lymphocyte sub-populations and the proliferative responses of splenocytes suggesting that these changes could be due to other factors such as gravity changes. Thus, CUMS, which is an easy to implement model, could contribute to deepen our understanding of some spaceflight-associated immune alterations and could be useful to test countermeasures.

## Introduction

Human bio-astronautic programs have substantially expanded over the last 50 years. Space is an adverse environment in which human encounter different types of stressors that can be classified in three categories: radiations, mechanical (microgravity and hypergravity), and socio-environmental (e.g., confinement, isolation, disrupted circadian rhythm…) stressors. Medical and physiological findings from these missions have demonstrated that this extreme environment negatively impacts almost all physiological systems. It causes muscle atrophy, bone demineralization, cardiovascular and metabolic dysfunctions, impaired cognitive processes and reduces immunological competence. Regarding this last point, it was shown that 15 of the 29 astronauts involved in Apollo missions developed bacterial or viral infections during, immediately after, or within 1 week of landing (Kimzey, 1977). In addition, the first study based on medical data collected on 46 astronauts who spent 6 months onboard the International Space Station, showed that 46% of them had to face immunological problems (Crucian et al., 2016a). These observations demonstrate that, on average, spaceflight affects the immune system of 50% of the astronauts and that immune dysregulations occurs during spaceflight, confirming in-flight dysregulation distinct from the influences of landing and readaptation following deconditioning (Crucian et al., 2015, 2016a,b).

There have been several studies to understand how spaceflight environment impairs innate immunity and T cell responses (reviewed in Guéguinou et al., 2009; Frippiat et al., 2016). It has been shown that the phagocytic and oxidative functions of neutrophils are affected by spaceflight conditions (Kaur et al., 2004; Rykova et al., 2008) and that astronauts’ monocytes exhibit phenotypic and cytokine-production deregulations, a reduced ability to engulf *E. coli*, elicit an oxidative burst and degranulate (Kaur et al., 2005, 2008; Rykova et al., 2008; Crucian et al., 2011). Low natural killer cell cytotoxicity and a delay in responses to hypersensitivity skin tests were observed (Taylor and Janney, 1992; Meshkov and Rykova, 1995). Reactivation of latent herpes viruses has frequently been reported and can be considered as a good biomarker of spaceflight-induced weakening of cell-mediated immunity (Mehta et al., 2000; Pierson et al., 2005; Cohrs et al., 2008). Numerous studies did also investigate reduced T cell activation under low gravity conditions (Cogoli et al., 1984; Cogoli, 1993a,b; Gridley et al., 2009) and highlighted that almost all cellular parameters can be affected such as : (i) genetic expression, as shown by lower expressions of Interleukin-2 (IL-2) and IL-2 receptor alpha chain (Walther et al., 1998); (ii) cell–cell interactions and cytoskeleton structure, as T lymphocytes were found to be highly motile under microgravity while the motility of monocytes was severely reduced and the structure of their cytoskeleton was modified (Sciola et al., 1999; Cogoli-Greuter, 2004; Meloni et al., 2004, 2006, 2011); (iii) signal transduction, as PKA and NF-κB signaling pathways were shown to contribute to T cell dysfunction under altered gravity (Boonyaratanakornkit et al., 2005; Chang et al., 2012; Martinez et al., 2015) and (iv) disturbed expression of cell cycle regulatory proteins (Thiel et al., 2012).

Humoral immunity has been less extensively studied, and inconsistent data were reported after long-term flights. Konstantinova et al. (1993) reported increased levels of serum IgA and IgG, while Rykova et al. (2008) indicated that the total amounts of serum IgA, IgG, and IgM were unchanged. Using the amphibian *Pleurodeles waltl* as an animal model (Frippiat, 2013), we previously showed that spaceflight affects antibody production in response to an antigenic stimulation (Boxio et al., 2005; Bascove et al., 2009). We also demonstrated that somatic hypermutations, that diversify antibody-binding sites to improve their affinity, occur following immunization in space but at a frequency two-times lower than on Earth (Bascove et al., 2011). Another space experiment, coupled with several ground-based simulations of stressors encountered during a mission onboard the ISS, demonstrated that the transcription of IgM heavy chains and of an early B cell transcription factor are modified only when embryos of *P. waltl* are subjected to gravitational changes, suggesting a change in B lymphopoiesis (Huin-Schohn et al., 2013).

Given the limitations in the availability and the experimental protocols that can be carried out with samples from astronauts as well as the cost and the limited number of space experiments, various ground-based models have been developed to reproduce the effects of spaceflight conditions on an organism. The most widely used to reduce gravity constraint are head-down tilt bed rest for humans (Hargens and Vico, 2016) and anti-orthostatic tail suspension for rodents (Globus and Morey-Holton, 2016), while continuous centrifugation of animals are used to increase gravitational force. Recently, we showed that hypergravity and simulated microgravity (anti-orthostatic suspension) impair the proliferative responses of murine lymphocytes (Guéguinou et al., 2012; Gaignier et al., 2014). Moreover, we showed that anti-orthostatic suspension induces a decrease of murine B lymphopoiesis, demonstrating that our hypothesis deduced from studies performed with *P. waltl* embryos that developed onboard the ISS was correct (Lescale et al., 2015). In the same way, gravitational changes were shown to affect T cell development and the repertoire of T cell receptors, suggesting that host immunity could be modified (Woods et al., 2003, 2005; Ghislin et al., 2015).

However, gravitational changes are not the only stressors encountered during space missions. Socio-environmental factors (e.g., confinement, isolation, circadian rhythm misalignment…) have to be considered because they can affect immune parameters (Choukèr, 2012; Frippiat et al., 2016). Here, to simulate socio-environmental stresses encountered during a space mission, we exposed adult male mice, as up to now most astronauts were males, to chronic unpredictable psychosocial and environmental stressors of various nature and mild intensity separated by resting periods (CUMS model). We chose this model, involving only mild stressors, because astronauts are heavily trained before flying and are enthusiastic to go to space. This positive rewarding effect, understandable after such long training, might compensate at least partially the negative effects of mission-associated stressors while for mice there is no rewarding effects. The second reason is that this model does not involve food and water deprivation, which is not something endured by astronauts either. The third reason is that CUMS involves resting periods to reflect the fact that ISS crew activities involve periods of notable stress (e.g., dockings, extra-vehicular activities) separated by less stressing periods. We therefore believed that this model comes reasonably close to the diversity and intensity of socio-environmental stressors encountered by astronauts while they are aboard the ISS (**Table 1**). Furthermore, it is easier to implement and more accessible than undersea deployment, Antarctic winter-over missions or Mars simulation.

**Table 1:** Comparison of socio-environmental stressors encountered during space missions with those delivered using the CUMS model, and limitations of this model.

Socio-Environmental stressors encountered during spaceflights	Socio-Environmental stressors applied in the CUMS model	Limitations of this model
Confinement throughout the mission.	Mice confined in a small cage during 1 or 2 h.	From an ethical point of view, mice cannot be confined during extended periods while astronauts are confined for several months in the ISS.
Isolation from friends and family.	Mice, which are sociable animals, were isolated during the whole CUMS procedure.	
Disrupted circadian rhythm.	15 h overnight period with permanent light + reversed light/dark cycle between Friday evening and Monday morning.	Astronauts observe 16 sunrises and sunsets during a 24 h period.
Crew tension and other interpersonal issues.	Pair housing during 2 h.	Pair housing is of a limited duration.
Perturbation of spatial references.	30° cage tilt for 1, 2, or 15 h.	Cage tilt is of a limited duration.
Lower dietary intake, despite enough available food, perhaps due to changes in eating habits and rituals.	15 h overnight period with difficult access to food, without a reduction in the daily food ration.	
Uncomfortable living conditions.	15 h overnight period in a soiled cage (mice do not like wet litter).	

Our data show that 3 weeks of CUMS exposure induces an increase of serum IgA, a reduction of normalized splenic mass and tends to decrease pro-inflammatory cytokine production, as previously reported during or after space missions. CUMS could therefore contribute to (i) deepen our understanding of some spaceflight-associated immune alterations and (ii) be useful to test countermeasures.

## Materials and Methods

### Animals

Experiments were conducted on 8-week-old C57Bl/6NCrl male mice with 20 g mean body weight purchased from Charles River Laboratories (Bois des Oncins, France). On arrival, animals were housed for 5 days in groups of five in standard cages in the animal facility of the INSERM UMR894 laboratory (Paris). Animals were provided food and water *ad libitum* in a quiet room with constant temperature (22°C), 50% relative humidity and 12 h light/dark cycles (dark period 8 pm–8 am). Then, animals were randomly divided in two groups: one control group and one group subjected to CUMS for 21 days. CUMS and control mice were housed in different rooms. Experiments were repeated twice. This study was carried out in accordance with the National Legislation and the Council Directive of the European Communities on the Protection of Animals Used for Experimental and Other Scientific Purposes (2010/63/UE). Moreover, the CUMS protocol was approved by the French Ministry of Research (authorization 00966.02).

### Chronic Exposure to Psychosocial and Environmental Stressors (CUMS)

Mice were isolated (one mice per cage) and subjected to six mild environmental or psychosocial stressors (**Table 1**): 30° cage tilt for 1 h, 2 h, or 15 h; confinement in a small cage (11 cm × 8 cm × 8 cm) for 1 h or 2 h; paired housing for 2 h; one 15 h overnight period with difficult access to food (without a reduction in the daily food ration); one 15 h overnight period with permanent light and one 15 h overnight period in a soiled cage (50 ml of water in 1,000 ml of bedding). These stressors were delivered according to Pardon et al. (2000). Stress periods that lasted 1 h in the morning, 2 h in the afternoon, and 15 h at night (6 pm–9 am) were always separated by stress-free intervals of at least 2 h to avoid any habituation process. Animals were also placed on a reversed light/dark cycle between Friday evening and Monday morning. This procedure was scheduled over a 1-week period and repeated throughout the 3 weeks of experimentation. The control group was left undisturbed in another room of the animal facility, five mice per standard cage (37.5 cm × 21.5 cm × 18 cm).

### Sample Collection

After 21 days of CUMS exposure, mice were weighed and put to death. Animals were killed between 8 and 10 am to avoid fluctuations of corticosterone concentration due to circadian rhythm. Trunk blood was collected, allowed to clot at ambient temperature for 15 min and centrifuged at 4°C and 4,000 rpm for 15 min to obtain serum samples that were stored at -80°C until analysis. The spleen and the thymus were also collected. The spleen was placed into sterile tubes containing 3 ml of RPMI 1640 medium (PAA, Pashing, Austria) to perform lymphocyte studies.

### Immunoglobulin Assays

The concentrations of serum immunoglobulins (IgA, IgG, and IgM) were determined using Mouse ELISA quantitation sets according to manufacturer instructions (Bethyl Laboratories Inc., Montgomery, TX, United States). Concentrations were calculated using a 4-parameter curve and expressed in μg/ml.

### Corticosterone Quantification

Corticosterone levels in serum samples were measured without any extraction procedure using the Corticosterone Enzyme Immunoassay kit (Arbor Assays, Ann Arbor, MI, United States). Concentrations were calculated using a 4-parameter curve and expressed in ng/ml.

### Lymphocyte Populations in the Spleen

Spleens were dissociated in RPMI 1640 medium. Red blood cells were lysed with NH_4_Cl 140 mM (eBioscience, San Diego, CA, United States) before counting nucleated cells with a hemocytometer. To evaluate lymphocyte sub-populations, 10^6^ splenocytes were incubated for 15 min at 4°C in the dark with a mixture of four anti-mouse monoclonal antibodies: ECD anti-CD19 (clone 6D5, Beckman Coulter, Marseille, France), APC anti-CD3𝜀 (clone 17A2, eBioscience), PE anti-CD4 (clone RM4-5, eBioscience) and PE-Cy7 anti-CD8α (clone 53-6.7, eBioscience). Immunophenotyping was carried out using a five-color FC500 flow cytometer (Beckman Coulter). Data were analyzed using the FlowJo v7.6.5 software (Tree Star Inc., Ashland, OR, United States).

### *In Vitro* Stimulations

Two mitogens, lipopolysaccharide (LPS) from *Escherichia coli* (Sigma-Aldrich) and concanavalin A (ConA) from *Canavalia ensiformis* (Sigma-Aldrich, St Louis, MO, United States), were used to stimulate splenocytes. Splenocytes were adjusted to 10^7^ cells/ml in RPMI 1640 culture medium supplemented with 10% heated FCS, 100 U penicillin, 100 μg/ml streptomycin and 2 mM glutamine (Sigma-Aldrich). Cells were then dispensed in 50 μl quadruplicates into a 96-well tissue culture plate containing 50 μl of culture medium without (unstimulated cells) or with (stimulated cells) mitogen at a final concentration of 5 μg/ml. This plate was incubated for 48 h at 37°C and 5% CO_2_. Then, 20 μl of MTS (Promega, Madison, WI, United States) was added into three wells to determine the number of viable cells. After 4 h of incubation at 37°C and 5% CO_2_, the optical density (OD) was measured at 490 nm and an index of proliferation (IP) was calculated using the following formula: IP = OD (490 nm) of stimulated cells/OD (490 nm) of unstimulated cells. The supernatant from the last well was frozen at -80°C for cytokine quantification.

### Quantification of Cytokines

Mouse sera and culture supernatants were thawed just before analysis. Cytokines (IFNγ, IL-12p70, IL-4, IL-5, IL-6 and TNFα) were quantified using the Bio-Plex^®^ Instrument (Bio-Rad, Ivry sur Seine, France) and the ProcartaPlex Mouse Essential Th1/Th2 Cytokine Panel (Affymetrix, Santa Clara, CA, United States). Concentrations were determined using the Bio-Plex^®^ software and expressed in pg/ml. The sensitivity of this kit is 0.09 pg/ml for IFNγ, 0.21 pg/ml for IL-12p70, 0.03 pg/ml for IL-4, 0.32 pg/ml for IL-5, 0.21 pg/ml for IL-6 and 0.39 pg/ml for TNFα.

### Statistics

SPSS v13.0 software (SPSS Inc., Chicago, IL, United States) was used to perform statistical analyses. Outlier values were determined by creating a boxplot for each studied group. Once normality and homogeneity of variances were assessed, as determined by Kolmogorov–Smirnov and Levene tests, an unpaired *t*-test was performed. When data were not normally distributed, a Mann–Whitney non-parametric test was performed. *p*-values < 0.05 indicate significance. *p*-values comprised between 0.05 and 0.10 indicate trend. All data are presented as means ± standard error of the means (SEM).

## Results

### Evaluation of Stress After 21 Days of CUMS Exposure

To assess the stress status of mice after 21 days of CUMS exposure, we first determined thymus and spleen weights and normalized these values to body weights (**Table 2**). No significant difference appeared between normalized thymus weights for the two groups of mice. However, a statistically significant reduction in normalized spleen weight was observed for CUMS mice (*p* = 0.0174). Finally, we quantified corticosterone, the most studied stress hormone in rodents. Our results show that its concentration was the same in the serum of mice exposed to 21 days of CUMS and control mice (**Table 2**).

**Table 2:** Body weights, lymphoid organ normalized weights and serum corticosterone concentration in control and CUMS mice.

	Body weight (g)	Normalized thymus weight (mg/g)	Normalized spleen weight (mg/g)	Corticosterone (ng/ml)
Control	25.38 ± 0.4	2.16 ± 0.09	2.97 ± 0.08	35.56 ± 6.5
CUMS	25.15 ± 0.4	2.08 ± 0.10	2.59^∗^ ± 0.06	35.64 ± 7.5

Normalized thymus and spleen weights were calculated using the formula: organ weight/body weight. Serum corticosterone concentrations were determined by ELISA. ^∗^*p* = 0.0174 versus control as determined using an unpaired ox*t*-test. *n* = 10. Results representative of two CUMS exposures. Data are presented as mean values ± SEM.

### Splenic Lymphocyte Populations

To understand why splenic mass is decreased in CUMS mice, we determined the number of nucleated cells in that organ. **Table 3** shows that this number was the same in both groups of mice. Furthermore, no statistically significant difference was observed between total lymphocyte percentages (**Table 3**).

**Table 3:** Lymphocyte sub-populations in the spleens of control and CUMS mice.

	Control	CUMS
Number of nucleated cells	97.5 ± 12.1	99.5 ± 7.4
% of total lymphocytes	69.6 ± 3.6	68.5 ± 2.9
% of CD19^+^ cells	39.5 ± 2.5	44.6 ± 1.5
% of CD3^+^ cells	52.0 ± 3.0	51.1 ± 1.5
% of CD3^+^ CD8^+^ cells	18.6 ± 1.3	19.6 ± 0.8
% of CD3^+^ CD4^+^ cells	31.0 ± 1.8	28.9 ± 0.8


Splenic nucleated cells were counted in CMUS (*n* = 6) and control (*n* = 8) mice after red blood cell lysis with NH_4_Cl and analyzed by flow cytometry. Results representative of two CUMS exposures. Data are shown as means ± SEM. No significant difference was found using an unpaired ox*t*-test.

To precise these data, we determined the percentages of major splenic lymphocyte sub-populations by immunophenotyping. CD19 was used to identify B cells, CD3 to identify T cells, CD3 + CD8 to identify cytotoxic T cells and CD3 + CD4 to identify helper T cells. Cells positive for each phenotypic marker, or a combination thereof, were quantified within the viable lymphocyte gate. Our data show that the percentages of CD19^+^ B cells, CD3^+^ T cells, CD3^+^ CD4^+^ helper T cells and CD3^+^ CD8^+^ cytotoxic T cells did not vary between the two experimental groups (**Table 3**). These results indicate that 3 weeks of CUMS exposure does not seem to modify major lymphocyte sub-populations in the spleen.

### Splenocyte Proliferative Response

**FIGURE 1:**
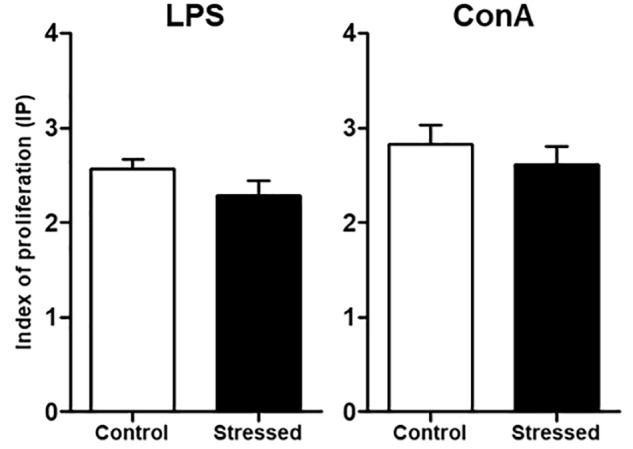
Proliferative responses of splenocytes from CUMS (*n* = 8) and control (*n* = 6) mice. After spleen dissociation in RPMI 1640 medium, cells were cultured during 48 h with LPS or ConA. Then, MTS was added and the optical density (OD) was measured at 490 nm to determine the index of proliferation (IP) using the formula: IP = OD (490 nm) of stimulated cells/OD (490 nm) of unstimulated cells. Results are shown as means ± SEM. No significant difference was found using an unpaired *t-*test. Results representative of two CUMS exposures.

**FIGURE 2:**
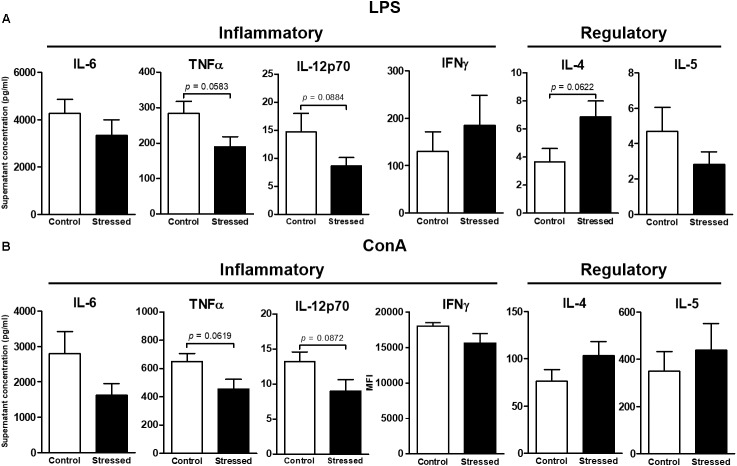
Cytokines secreted by splenocytes of CUMS (6 ≤ *n* ≤ 7) and control (4 ≤ *n* ≤ 5) mice after 48 h of *in vitro* stimulation with LPS **(A)** or ConA **(B)**. The concentrations of six Th1/Th2 cytokines in culture supernatants were determined using the ProcartaPlex^TM^ kit and the Bio-Plex^®^ instrument. Data are shown as means ± SEM. In **(B)**, MFI (mean fluorescence intensity) values are presented for IFNγ because values were outside the standard curve established using the mixture of antigen standards provided by the manufacturer. Tendencies to significant differences were found using unpaired *t*-tests. Results representative of two CUMS exposures.

**FIGURE 3:**
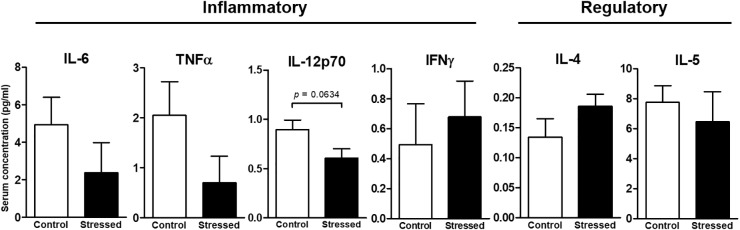
Concentrations of six Th1/Th2 cytokines in the serum of CUMS (6 ≤*n* ≤ 7) and control (*n* = 5) mice. Quantifications were performed using the ProcartaPlex^TM^ kit and the Bio-Plex^®^ instrument. Data are shown as means ± SEM. Tendency to significant difference was found for IL-12p70 using an unpaired *t*-test. Results representative of two CUMS exposures.

We then questioned whether splenic immune cells proliferative responses could be affected by 21 days of CUMS exposure. To address this question, splenocytes were stimulated with LPS or ConA during 48 h. This was the minimal duration to observe a proliferative response according to our previous study (Guéguinou et al., 2012). Our results show that the LPS- and ConA-induced proliferative responses were not different between the two groups of mice (**Figure 1**). Thus, these responses were not affected after 3 weeks of CUMS exposure. We also quantified a panel of six Th1/Th2 cytokines into the culture medium following these stimulations (**Figure 2**). Our results revealed trends toward a reduction of pro-inflammatory cytokines production by CUMS splenocytes following both stimulations. Interestingly, IL-12p70 concentration also tended to be lower in the sera of CUMS mice (*p* = 0.0634) (**Figure 3**). Taken together, these data suggest that CUMS exposure could reduce the production of pro-inflammatory cytokines.

### Serum Immunoglobulins

**FIGURE 4:**
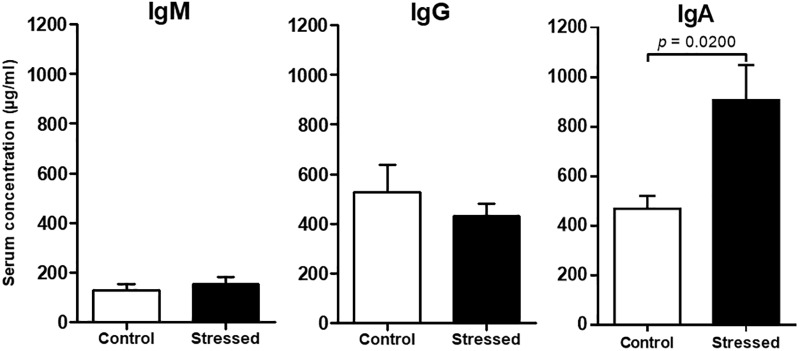
Immunoglobulin concentrations in the sera of CUMS (*n* = 8) and control (*n* = 6) mice determined by ELISA. Histograms represent mean ± SEM. Differences were found to be statistically significant using a Mann–Whitney test for IgA. No significant difference was found using an unpaired *t*-test for IgG and IgM. Results representative of two CUMS exposures.

Finally, we quantified major antibody isotypes in mouse sera because previous studies suggested that spaceflight could affect their expression (Konstantinova et al., 1993; Boxio et al., 2005). Our analyses revealed that IgM and IgG concentrations were not significantly modified in the serum of CUMS mice (**Figure 4**). However, IgA concentration was significantly increased (*p* = 0.0200).

## Discussion

The aim of this study was to determine if a model involving the chronic exposure of mice to multiple unpredictable mild environmental and psychosocial stressors, CUMS, could replicate some immune changes reported during or after space missions. Such easy to implement model would be highly desirable given the limited access to undersea deployment, Antarctic winter-over missions or Mars simulation experiments. Furthermore, using mice would overcome the limitations in the availability and the experimental protocols that can be carried out with human beings.

We first noted that after 3 weeks of CUMS exposure, a duration chosen to simulate a long flight at the human scale, mice did not present an activation of the hypothalamic-pituitary-adrenal (HPA) axis. Similarly, ISS crew generally adapt to the space environment (Crucian et al., 2016b). Usually, urine cortisol appears elevated early during flight, but returns to baseline following adaptation (Crucian et al., 2015).

A decrease of normalized splenic mass of 1.2 times was observed in CUMS mice. Similarly, previous studies have shown that spaceflight conditions reduce murine splenic mass by a 1.3 factor (Gridley et al., 2003; Baqai et al., 2009). However, CUMS did not modify splenic nucleated cells numbers and major lymphocyte sub-populations while changes in rat and mice splenic lymphocyte sub-populations were reported after spaceflight (Sonnenfeld et al., 1992; Pecaut et al., 2003; Baqai et al., 2009; Gridley et al., 2009). This difference is likely due to the fact that spaceflight induce a redistribution of body fluids (Vernikos, 1996) which is unlikely with CUMS. CUMS-induced lower splenic mass could result from a decrease in erythropoiesis because this physiological process was shown to be modulated by chronic psychosocial stress (Vignjevic et al., 2015). Interestingly, spaceflight conditions were also reported as having a negative impact on erythropoiesis (Davis et al., 1996).

As indicated in the introduction of this paper, reduced T cell responses were reported after spaceflight. Less information is available about the effects on B cell responses but they are likely reduced too because less somatic hypermutations were noted in immunoglobulin heavy chains after inflight immunization (Bascove et al., 2011). It was also shown that B and T cell proliferative responses are reduced when mice were subjected to gravity changes (2G hypergravity or anti-orthostatic suspension) during 3 weeks (Guéguinou et al., 2012; Gaignier et al., 2014). In contrast, the responses of these lymphocytes were not modified after 3 weeks of exposure to socio-environmental stressors (LPS and ConA are known to stimulate murine B and T cells, respectively). These data suggest that spaceflight-inducted lower lymphocyte responsiveness mainly result from gravity changes. This conclusion is in agreement with another study having shown that changes in gravity (hypergravity and microgravity) affect the expression of IgM heavy chain mRNAs, whereas other stressors encountered during a mission (radiation, confinement, disruption of the circadian rhythm, heat shock encountered during atmosphere re-entry onboard a Soyuz vehicle) did not (Huin-Schohn et al., 2013).

Serum cytokine levels were low in mice exposed to CUMS. This pattern is consistent with mice free of infection. A slight reduction of serum IL-12p70 concentration (1.5 times less) was noted in CUMS mice. This decrease of pro-inflammatory cytokine production appeared also after *in vitro* stimulation of splenocytes. Indeed, both IL-12p70 and TNFα levels tended to decline by an average 1.5 factor in culture medium after ConA and LPS stimulations. These statistical trends are perhaps due to the fact that after 48 h of stimulation we were not at the best moment to quantify cytokines. Furthermore, cytokine concentrations were shown to fluctuate during spaceflight (e.g., Crucian et al., 2015). Interestingly, it was recently shown that the levels of 10 out of 11 inflammatory or adaptive/regulatory cytokines remained low in the sera of astronauts during long-duration spaceflights (6 months) (Crucian et al., 2014). Nevertheless, when peripheral blood mononuclear cells of crewmembers involved in a long-duration mission in the ISS were stimulated with anti-CD3 and anti-CD28 to activate T cells or with PMA and ionomycin as a broader stimulus, these cytokines concentrations in culture supernatants were also decreased by an average 1.5 factor when comparing values obtained before launch with those determined on landing day (Crucian et al., 2015). Here, we did not observe a decline in adaptive/regulatory cytokine secretion. This difference could perhaps be because our mice experienced only a combination of various mild psychosocial and environmental stressors, while crewmembers additionally experienced microgravity. It may also be possible that mice adapt more rapidly or easily than human beings to stressful situations. Whatever the reasons are, these two independent studies are in agreement with results presented here.

Finally, we observed that the concentration of IgA was two times higher in the serum of CUMS mice. Such increase of IgA concentration was also noted in stressed people (Viena et al., 2012), in adult *Pleurodeles waltl* after 6 months spent in the Mir space station (Boxio et al., 2005), mice exposed to hypergravity (Guéguinou et al., 2012) and people who faced 1 month of extreme Antarctic conditions (Mishra et al., 2012; Yadav et al., 2012). Together, these data suggest that IgA could be used as a stress biomarker.

## Conclusion

This study shows that CUMS exposure increases serum IgA concentration, reduces spleen weight and tends to reduce the production of pro-inflammatory cytokines, as previously reported during or after space missions. However, this model does not reproduce spaceflight-induced modifications of splenic lymphocyte sub-populations and the lower responsiveness of lymphocytes. These data show that CUMS, which is an easy to implement model, could contribute to deepen our understanding of some spaceflight-induced immune alterations and could be useful to test countermeasures. In the future, it would be interesting to (i) perform a kinetic evaluation of immunological changes during CUMS exposure because physiological and immune changes may be dynamic and adaptation processes, despite resting periods included in the CUMS protocol, could be active during the experiment, (ii) examine the effects of CUMS exposure on specific lymphocyte subsets and (iii) submit latently infected mice to CUMS to see if this model could induce the reactivation of latent herpes viruses as frequently reported in astronauts.

## Data Accessibility

Datasets generated for this study are included in the manuscript.

## Author Contributions

J-PF, CC-S, and LL conceived/designed the experiments. FG, CL-F, ES, JM, and J-LM performed the experiments. FG and CL-F analyzed the data. J-PF, FG, and CL-F wrote the manuscript in conjunction with other authors. All authors contributed to manuscript revision, read, and approved the submitted version.

## Conflict of Interest Statement

The authors declare that the research was conducted in the absence of any commercial or financial relationships that could be construed as a potential conflict of interest.

## Abbreviations

CUMS: chronic unpredictable mild psychosocial and environmental stressors
